# Could Different Eubiotics Improve Gut Health, Growth Performance, Carcass Yield, and Skin Pigmentation in Broilers Fed Sorghum–Soybean-Based Diets?

**DOI:** 10.3390/ani16121838

**Published:** 2026-06-15

**Authors:** Osiris Napoleón Pérez-Segura, Arturo Cortés-Cuevas, Gabriela Gómez-Verduzco, Ernesto Avila-González

**Affiliations:** 1 Centro de Enseñanza, Investigación y Extensión en Producción Avícola CEIEPAv, Facultad de Medicina Veterinaria y Zootecnia, Universidad Nacional Autónoma de México, Tláhuac, Ciudad de México 13300, Mexico; mvz.operez@gmail.com (O.N.P.-S.); cortescuevasarturo@yahoo.com (A.C.-C.); avilaernesto@yahoo.com (E.A.-G.); 2 Departamento de Medicina y Zootecnia de Aves, Facultad de Medicina Veterinaria, Universidad Nacional Autónoma de México, Ciudad Universitaria, Ciudad de México 04510, Mexico

**Keywords:** eubiotics, probiotics, phytobiotics, productive performance, intestinal health

## Abstract

The objective of this research was to evaluate different commercial eubiotics. The eubiotics were evaluated individually and in combination on growth performance, carcass traits, and gut structure in broilers fed sorghum–soybean meal diets. A total of 1000 Ross 308 male broilers were randomly allocated to one of five dietary treatments with eight replicates of 25 broilers each. The experimental treatments were: 1. CON (basal diet), 2. ENR (basal diet + Enradin^®^ 100 g/ton), 3. PF (basal diet + Probion-forte© 300 g/ton), 4. PF+ EB (basal diet + Probion-forte© and EndoBan FT^®^ 250 and 250 g/ton, respectively), 5. CPP (basal diet + CRINA^®^ Poultry Plus 300 g/ton). Broilers fed with eubiotic-supplemented diets showed significantly ameliorated growth performance compared with the control group (CON *p* < 0.05). Carcass weight was also significantly higher in broilers fed with eubiotics than those fed CON (the basal diet, *p* < 0.05). Gut structure analysis showed an increased villus height and mucosal thickness in the duodenum of eubiotic-treated groups (*p* < 0.05). The mixed supplementation of Probion-forte© and EndoBan^®^(PF-EB) induced greater villus height and mucosal thickness in the jejunum and ileum (*p* < 0.05). Additionally, supplementation with PPR (CRINA^®^ Poultry Plus) increased crypt depth in the duodenum and jejunum (*p* < 0.05), whereas PF (Probion-forte©) improved the villus height-to-crypt depth ratio in the duodenum and ileum (*p* < 0.05). Consequently, dietary supplementation with these commercial eubiotics, individually and in combination, could ameliorate productive performance, carcass yield, skin pigmentation, and gut structure in broilers.

## Simple Summary

Consumers worldwide are exerting significant pressure to demand healthier and more natural food production; the poultry meat industry is particularly exposed to this high demand. Consequently, numerous natural alternatives have emerged to improve the poultry industry. This perspective was the basis of this research, which tested various commercial eubiotics with different mechanisms of action in a broiler chicken diet. Treatments with eubiotics showed statistically significant differences in live weight, weight gain, and feed conversion ratio. No differences were found between treatments in the percentage of hot and chilling carcass yield; however, statistically significant differences were observed in carcass weight in the eubiotics treatments compared to the control group. Similarly, improvements were found in the height of villi, thickness, and depth of the duodenal and jejunal crypts with some of the eubiotics tested compared to the control group. The different commercial eubiotics showed a beneficial effect on production parameters, carcass yield, and pigmentation, as well as an improvement in intestinal health.

## 1. Introduction

Poultry has undergone an unprecedented transformation. Modern broiler chickens have been genetically selected for exceptional growth efficiency, supported by a high metabolic rate that enables rapid muscle accretion within a short production cycle. While this genetic progress in poultry farming has notably impacted productivity, its effect on the gastrointestinal tract [1] is also remarkable; optimal intestinal functionality is a critical determinant for productive performance, host resistance, and overall sustainability. The gastrointestinal tract in broilers has been considered a very important anatomical structure physiologically, where nutrient digestion, immune responses, and microbiome balance (eubiosis) happen. Eubiosis has been reported in health conditions. Balanced and functionally stable intestinal microbiota support epithelial integrity, efficient nutrient absorption, and an appropriate immune function [2,3].

Conversely, disruptions to this equilibrium may lead to dysbiosis, characterized by impaired barrier function, reduced digestive efficiency, and increased metabolic costs associated with immune stimulation.

When gut structure and microbiome homeostasis are compromised, nutrient digestion and absorption are directly affected, resulting in suboptimal growth performance and increased nutrient excretion. This inefficiency not only reduces economic returns but also exacerbates environmental pressure through the loss of nitrogen- and phosphorus-rich nutrients that require substantial resources to produce [4]. Nutritional strategies have been used to maintain intestinal eubiosis; maximum optimization of nutrient absorption has become a central focus in poultry production systems.

Different kinds of eubiotics have gained relevance as functional feed additives [5,6]. Interestingly, their action mechanisms are different; for example, some function by improving intestinal integrity, growth performance, or improving the microbiome.

Probiotics, members of the genus *Bacillus*, have received considerable attention due to their ability to form endospores, which confer thermal stability and resistance to acidic environments, allowing them to survive [7]. It has also been reported to improve the production of extracellular enzymes at the intestinal level and improve the absorption surface villi [8]. Lactobacillus spp. has demonstrated acidification of the intestinal lumen by lactic acid and protection of tight junctions [9]. Other bacterial species, such as *Clostridium butyricum*, have also demonstrated the capacity to enhance butyric acid production in the ceca, thereby supporting epithelial integrity, tight junction function, and improving nutrient absorption [10,11].

Commercially available other mixtures of nutritional compounds are based on (a) clay minerals such as bentonites or aluminosilicates that trap endotoxins (lipopolysaccharides or LPS) through electrostatic forces; (b) red algae extracts, which enhance the binding capacity of bacterial toxins; (c) phytogenic compounds represented by mixtures of plant extracts (such as flavonoids) that stimulate natural enzymes like intestinal alkaline phosphatase (ALP), which chemically break down toxins to neutralize them [12]. 

Other phytogenic feed additives are the plant-derived bioactive compounds thymol, eugenol, and piperine, which have also been used as eubiotics. For example, thymol has been shown to increase intestinal villi height and stimulate pancreatic amylase and protease activity [13,14].

Eugenol reduces pro-inflammatory cytokines and increases serum superoxide dismutase (SOD) levels [15,16]. Piperine inhibits P-glycoprotein and metabolic enzymes, facilitating nutrient absorption; it modulates intestinal transit time and improves xanthophyll absorption by facilitating transport across the mucosa [17,18].

Tiihonen et al. (2010) [19] suggest that the use of these three molecules in a blend produces a synergistic effect superior to that of each one separately.

Based on the premise that different classes of eubiotics promote intestinal health through distinct biological pathways, it was hypothesized that dietary supplementation with probiotics, phytogenic compounds, and organic acids, individually and in combination, would result in differential effects on growth performance, intestinal integrity, carcass yield, and skin pigmentation redness in broilers.

## 2. Materials and Methods

### 2.1. Experimental Site and Ethical Approval

All the procedures involving broiler chickens used in this study were approved by the Institutional Animal Care and Use Committee (Comité Institucional para el cuidado y uso de los animales experimentales-CICUAE FMVZUNAM) and conducted according to Official Mexican Norm (NOM-033-SAG/ZOO-2014) guidelines for animal welfare and experimental protocols under approval number SICUAE.MC-2022/3-3. This research was carried out at the CEIEPAV facilities located in Manuel M. López s/n, Santa Ana Poniente, Tláhuac, 13300 CDMX, Mexico.

### 2.2. Bird Husbandry, Experimental Design, and Diets

A total of 1000 one-day-old (Ross 308) broiler chicks of both sexes (50:50 ratio; Aviagen North America, Huntsville, AL, USA) were obtained from a commercial hatchery in a 42-day experimental period. Birds were randomly distributed into 40 floor pens with 25 birds per pen, distributed in 5 treatments with 8 replicates each. The floor space per pen was 3 m^2^ (0.12 m^2^/bird; 8.3 aves/m^2^). Each pen was equipped with wood shavings as bedding material at a depth of 5 cm. The housing facility relied on natural ventilation, with thermal comfort maintained through manual curtain adjustments based on bird age and ambient conditions. The temperature was gradually reduced from 32 °C on day one to 21 °C by day 28.

The experimental design comprised five treatments as follows:

1. Treatment **CON**: sorghum, soybean meal, basal diet
2. Treatment **ENR**: Basal diet + 10 ppm of enramycin; antibiotic growth promoter (100 g/ton. Enradin^®^ F80 MSD) Animal Health is a division of Merck & Co., Inc., Kenilworth, NJ, USA);
3. Treatment **PF**: Basal diet + 30 ppm of spore-forming probiotic; Probion Forte^®^ (300 g/ton. Woogene B&G, Seoul, Republic of Korea) containing *Bacillus subtilis* 1 × 10^8^ UFC/g, *Bacillus coagulans* 1 × 10^8^ UFC/g y *Clostridium butyricum* 1 × 10^6^ UFC/g;
4. Treatment **PF + EB**: Basal diet + spore-forming probiotic; Probion Forte^®^ (250 g/ton) + endotoxin adsorbent; EndoBan FT^®^ (Nutrex, Olen, Belgium, specific silicates, mixture of aromatic substances, and red algae; 250 g/ton);
5. Treatment **CPP**: Basal diet + gut health–promoting feed additive CRINA^®^ Poultry Plus (300 g/ton; benzoic acid, thymol, eugenol, piperine DSM-firmenich, Kaiseraugst, Switzerland).

All diets were based on sorghum–soybean meal (Table 1); water and feed were provided ad libitum. All additives were thoroughly mixed into the feed to ensure homogeneous distribution.

### 2.3. Productive Performance

Broilers and feed were weighed weekly for 42 days, and the weight gain, feed intake, and feed conversion ratio were obtained. Mortality was recorded daily. Carcass yield and skin Pigmentation were evaluated at 42 days.

### 2.4. Gut Structure

At 42 days of age, eight birds per treatment were humanely euthanized in accordance with NOM-033-SAG/ZOO-2014 regulations for intestinal morphometric evaluation. Approximately 2 cm segments were collected from the duodenum (midpoint of the duodenal loop), jejunum (5 cm proximal to Meckel’s diverticulum), and ileum (2 cm proximal to the cecal junction). Intestinal contents were gently removed using distilled water to clean the tissue samples.

Tissue samples were fixed by intraluminal perfusion followed by immersion in 10% buffered formalin (pH 7.2). Subsequently, samples were processed using standard histological procedures, embedded in paraffin, sectioned, and stained with hematoxylin and eosin [20].

Histological sections were examined at a total magnification of 40× using a light microscope (Motic BA310^®^, Hong Kong, China; Leica Microsystems, Schweiz AG, CH-9435 Heerbrugg, Switzerland) equipped with a digital microscopy camera and connected to a computer. Images were analyzed using Leica LAS EZ software (Leica Application suite) Version 3.3.0.

Villus height and crypt depth were measured longitudinally from the base to the tip of each structure and expressed in micrometers (μm). Five to six microscopic fields per histological section were evaluated to determine the number of villi present in each field within the duodenum, jejunum, and ileum. The villus height-to-crypt depth ratio (VH) was calculated by dividing villus height by crypt depth. Total mucosal thickness was calculated as the sum of villus height and crypt depth.

### 2.5. Carcass Yield and Skin Pigmentation

At 42 days of age, 25 broilers per treatment were fasted for 8 h prior to slaughter and individually weighed. Afterward, they were electrically stunned and slaughtered by exsanguination. Scalding at 53 °C for 1 min, mechanical defeathering, and manual evisceration. Hot eviscerated carcass weights (without head and feet) were recorded to calculate carcass yield.

Skin pigmentation of the carcasses was evaluated using a reflectance colorimeter (Minolta CR-400, Tokyo, Japan). Redness (a*) and yellowness (b*) values were determined in accordance with the CIELab color space system.

### 2.6. Statistical Analysis

The results of the variables obtained were analyzed to verify compliance with the assumptions of normality of the residuals using the Shapiro–Wilk W test and the homogeneity of variances with the Levene test, setting a significance level of 5% for both tests [21].

The data collected were analyzed using analysis of variance (ANOVA) to determine the effect of dietary treatments. When significant differences among treatments were detected (*p* < 0.05), means were compared using Tukey’s multiple comparison test. Statistical significance was established at *p* < 0.05. Pearson´s correlation analysis was perfomed to determine the the relationships among weight gain, carcass weight, and villus height-to-crypt depth ratios in the duodenum, jejunum, and ileum. R software Version 4.3.2 (R Foundation for Statistical Computing, Vienna, Austria, 2023).

## 3. Results

### 3.1. Productive Performance

The effects of dietary treatments on growth performance parameters at 42 days of age are presented in Table 2. Significant differences were observed among treatments for body weight gain (*p* < 0.05). Broilers fed diets supplemented with ENR, PF, PF + EB, and CPP showed greater weight gain compared with the control group. The highest values were recorded in the ENR (2331.4 g) and PF (2326.4 g) treatments, followed by CPP (2312.6 g) and EB + PF (2308.0 g), whereas the control group exhibited the lowest weight gain (2230.4 g).

Feed conversion ratio was also affected by dietary supplementation (*p* < 0.05). All supplemented treatments showed improved feed efficiency compared with the control, with lower feed conversion values observed in ENR and PF (1.65), followed by EB + PF and CPP (1.66). In contrast, the control treatment presented the highest feed conversion ratio (1.69).

No significant differences were detected among treatments for feed intake or mortality rate (*p* > 0.05).

### 3.2. Gut Structure

The effects of dietary treatments on villus height and crypt depth in the duodenum, jejunum, and ileum at 42 days are summarized in Table 3.

In the duodenum, villus height differed among treatments (*p* < 0.05). The control group exhibited the shortest villi (2028 µm), whereas all supplemented treatments showed greater villus height, with the highest value observed in PF (2354 µm), followed by PF + EB (2227 µm), CPP (2221 µm), and ENR (2203 µm). Crypt depth also differed among treatments (*p* < 0.05), with the greatest depth observed in CPP (377 µm), followed by PF + EB (335 µm) and PF (306 µm). The lowest crypt depth values were recorded in ENR (287 µm) and CON (293 µm).

In the jejunum, significant differences in villus height were observed among treatments (*p* < 0.05). The control treatment exhibited the shortest villi (1091 µm), whereas the EB + PF treatment showed the greatest villus length (1263 µm), followed by PF (1210 µm), CPP (1191 µm), and ENR (1160 µm). Crypt depth also varied among treatments (*p* < 0.05), with the greatest depth observed in CPP (268 µm). Intermediate values were recorded for CON (239 µm), PF (245 µm), and PF + EB (245 µm), while ENR exhibited the lowest crypt depth (226 µm).

In the ileum, villus height differed among treatments (*p* < 0.05). The control group showed the shortest villi (809 µm), whereas the PF + EB treatment exhibited the greatest villus height (909 µm). Intermediate values were observed in ENR (858 µm), CPP (865 µm), and PF (870 µm). Crypt depth also differed significantly (*p* < 0.05), with the lowest value recorded in PF (190 µm) and the highest in ENR (230 µm).

#### 3.2.1. Ratio Villus Height: Crypt Depth Ratio

The villus height-to-crypt depth ratios for the different intestinal segments are presented in Table 4. In the duodenum, significant differences were observed among treatments (*p* < 0.05). The highest ratios were recorded in PF (7.97) and ENR (7.79), followed by CON (7.10) and PF + EB (6.89), whereas CPP showed the lowest ratio (6.03).

In the jejunum, the PF + EB treatment exhibited the highest ratio (5.40), followed by ENR (5.19) and PF (5.14). Lower ratios were observed in CPP (4.69) and CON (4.68) (*p* < 0.05).

In the ileum, significant differences were also detected (*p* < 0.05). The lowest ratio was observed in ENR (3.82), whereas PF showed the highest ratio (4.85). Intermediate values were recorded for CON (4.09), CPP (4.25), and PF + EB (4.56).

The effects of dietary treatments on total intestinal mucosa thickness are shown in Table 5. In the duodenum, significant differences were observed among treatments (*p* < 0.05). Greater mucosal thickness was recorded in PF + EB (2562 µm), PF (2560 µm), CPP (2535 µm), and ENR (2490 µm) compared with the control treatment (2321 µm).

In the jejunum, mucosal thickness also differed among treatments (*p* < 0.05). The PF + EB treatment showed the greatest thickness (1478 µm), followed by PF (1455 µm) and CPP (1453 µm). Lower values were observed in ENR (1385 µm) and CON (1331 µm).

In the ileum, significant differences were detected as well (*p* < 0.05). The control group presented the thinnest mucosa (1016 µm), whereas the PF + EB treatment showed the greatest thickness (1106 µm). Intermediate values were recorded for PF (1060 µm), CPP (1075 µm), and ENR (1101 µm).

To further explore the relationships among gut structure, weight gain, and carcass yield outcomes, Pearson correlation coefficients were calculated. There was no correlation (*p* >0.05).

#### 3.2.2. Carcass Yield and Skin Pigmentation

Average carcass weights and yield percentages at 42 days are shown in Table 6. Carcass yield percentage was not affected by dietary treatments (*p* > 0.05). However, significant differences were observed in slaughter weight (*p* < 0.05). Broilers from the PF (1918.0 g), ENR (1915.2 g), PF + EB (1910.5 g), and CPP (1883.8 g) treatments achieved higher carcass weights compared with the control group, which recorded the lowest value (1773.2 g).

Skin pigmentation results for live birds and carcasses are presented in Table 7. Redness (a*) values of live skin did not differ among treatments (*p* > 0.05). In contrast, carcass skin redness was significantly influenced by dietary supplementation (*p* < 0.05). The highest redness value was observed in the PF treatment (4.63), followed by ENR (3.70) and PF + EB (3.05). Lower redness values were recorded in the CON (2.68) and CPP (2.18) treatments.

No significant differences were detected among treatments for yellowness (b*) values in either live skin or carcass skin (*p* > 0.05).

## 4. Discussion

### 4.1. Productive Performance

During the experimental period (1–42 days), broiler chickens that received supplemented diets with commercial eubiotics (PF and PF + EB) achieved weight gain and feed conversion ratios comparable to those observed in broilers fed the antibiotic growth promoter enramycin (ENR). These results indicated that the evaluated eubiotics were able to maintain productive performance at levels like those obtained with the growth antibiotic previously used, supporting their potential as effective alternatives in broiler nutrition.

Sultan A. et al. (2024) [22] reported similar responses with *Bacillus subtilis* in growth performance with those reported in this study. Likewise, [10] Zhang Z. et al., 2018 demonstrated that supplementation with Bacillus subtilis at 750 g/ton significantly improved daily weight gain and feed conversion. According to this, [23] Beyari et al. (2024) and [24] Khan S. et al. (2025) reported that *Bacillus pumilus* supplementation not only enhanced weight gain and feed efficiency but also exerted positive effects on liver function and immune response in broiler chickens. Hussain et al., 2024 [25] demonstrated that the administration of probiotics during the first 10 days was sufficient to induce improvements in productive performance, similar to those observed in broilers fed with growth-promoting antibiotics.

Regarding the phytogenic additive PPR treatment, broilers showed a 3.7% increase in weight gain and an improvement in feed conversion (1.67 vs. 1.70) compared with the control treatment (CON). These enhancements could be associated with documented effects of essential oils on gastrointestinal function, particularly their capacity to support a balanced intestinal microbiota “eubiosis” and enhance nutrient utilization [17]. In agreement with this interpretation, Huang et al. (2025) [26] reported that the inclusion of essential oils in broiler chickens’ diets positively influenced both productive performance and intestinal morphology. However, Jabbar et al. (2024) [27] reported that the inclusion of peppermint essential oil affected weight gain.

### 4.2. Gut Structure

The increase in villus height, villus height/crypt depth ratio, and intestinal mucosal thickness are important indicators of intestinal health, as the gut plays a central role in nutrient digestion, absorption, and protection against pathogens and toxins [28]. In this study, broilers supplemented with probiotic strains ameliorated intestinal morphology compared with the control treatment, which may have contributed to the better productive performance observed. Similar findings have been reported by Valipourian et al. (2025) [29] and Rivera et al. (2021) [30].

The greater villus height observed in the eubiotic-treated groups suggests an increased absorptive surface area, which may be associated with the improvements in body weight and feed conversion ratio. Although some treatments also showed increased crypt depth, this finding should be interpreted with caution, as crypt depth alone does not provide direct information about the underlying physiological processes. Therefore, the observed changes in intestinal morphology indicate a potential positive effect of eubiotic supplementation on intestinal architecture, although further studies are needed to clarify the mechanisms involved.

The use of phytobiotics and probiotics promotes an increase in the villi height, intestinal, and a decrease in the depth of the crypt, indicating mature, healthy, and functionally active epithelia, capable of greater absorption of available nutrients. In contrast, shorter villi would decrease the absorption surface of the nutrients and therefore the productive performance of broilers [31]. Rivera et al. 2021 [30], therefore, the depth of the crypts reflects the differentiation activity of the enterocytes, being responsible for cell proliferation throughout the intestine; the increase in the depth of intestinal crypts is associated with a better nutritional expenditure for the maintenance of the intestine, decreasing the absorption of nutrients and therefore productive efficiency [32].

### 4.3. Carcass Processing

In this study, statistical differences were observed in live weight and relative carcass weights in chickens fed with the various commercial eubiotics and enramycin. Rehman et al. (2021) [33] obtained similar results to those reported in this study, with no significant differences in carcass yield percentage, carcass weight, breast weight, and thigh weight.

Several studies that have evaluated carcass characteristics in broiler chicken fed diets supplemented with probiotics report that improve meat quality and flavor by improving amino acid and fatty acid profiles, significantly increasing breast muscle and therefore decreasing abdominal fat, in addition to improving water retention capacity, tenderness, sensory properties, and microbial safety (Tang et al., 2021) [34].

The analysis of productive performance and carcass yield in the case of the phytobiotic (CPP treatment) showed 6.0% live weight, 6.6% hot carcass weight, and 6.2% cold carcass weight; superior to the control group treatment (CON). The PF (probiotic) treatments showed 6.7% live weight, 8.2% hot carcass weight, and 8.2% cold carcass weight, and the PF + EB treatments were 6.8%, 8.0%, and 7.7%, respectively. Similar results in terms of low productive performance for some phytobiotics were reported by Ding et al. (2020) [35], who evaluated star anise in two different presentations (essential oil and leaves) and found no statistically significant differences in live weight and carcass yield compared to the control treatment, which was also reported by Moreno et al. (2021) [36] who evaluated moringa leaf powder and agave inulin.

The improvement in body weight may be associated with enhanced nutrient utilization and growth performance; however, the absence of changes in carcass yield suggests that the additional weight gain was distributed proportionally among carcass tissues and visceral organs. This indicates that the dietary treatments improved growth rate rather than selectively promoting muscle deposition.

Carcass pigmentation has not generally been evaluated in research conducted on broilers with eubiotic supplementation in the diet, possibly due to the low importance of skin pigmentation in broiler chickens in some countries; however, this carcass pigmentation is important to some consumers, associate bright yellow skin with freshness and quality Avila E. et al. (2000) [37] reported a tendency for the color of the carcasses of broilers fed with *Bacillus toyoi*. Alqahtani, F. et al. 2024 and Xu, L. et al. (2017) [38,39] demonstrated that the inclusion of *Bacillus pumilusinin* and *Bacillus coagulans* in broilers improved skin yellowing. Further research is needed using different eubiotics, individually or in combinations, evaluating diets with different grain bases (corn, wheat, etc.). This research should elucidate whether there is synergy, potentiation, or upregulation in these combinations, as well as the molecular mechanisms involved and their effect on sustainability.

## 5. Conclusions

The inclusion of the evaluated commercial eubiotics, including a spore-forming probiotic (Probi-on-forte^®^), an endotoxin adsorbent combined with probiotics (Probion-Forte^®^ + EndoBan FT^®^), and a blend of benzoic acid, thymol, eugenol, and piperine (CRINA^®^ Poultry Plus), in sorghum–soybean meal diets was associated with improved live weight, weight gain, feed conversion ratio, and hot and chilled carcass weight in broiler chickens from 1 to 42 days of age. Under the conditions of this study, these responses were comparable to those observed with the ENR treatment, suggesting that these eubiotics may represent a promising nutritional alternative.

The evaluated eubiotics were also associated with changes in intestinal morphology, including greater villus height, crypt depth, mucosal thickness, and villus height-to-crypt depth ratio in the duodenum, jejunum, and ileum. However, these morphological changes were not correlated with weight gain or carcass yield. Further studies are needed to better understand the relationship between intestinal morphology and productive performance, as well as the mechanisms involved in the response to these eubiotics when used individually or in combination.

## Figures and Tables

**Table 1:** Composition and calculated analysis basal starting, growth and finisher diets for broilers chickens.

Ingredient	Starting (0–10 Days)	Growth (11–21 Days)	Finisher (22–42 Days)
Sorghum	**632.171**	**650.749**	**670.469**
Soybean meal	**321.574**	**297.508**	**262.796**
Calcium carbonate	**19.304**	**18.234**	**17.702**
Soybean oil	**8.81**	**15.174**	**28.677**
Pigment	**-----**	**-----**	**6.00**
Orthophosphate	**6.12**	**3.43**	**1.81**
DL-Methionine	**3.01**	**3.13**	**2.74**
Salt	**2.94**	**3.03**	**3.10**
Vitamin and Mineral Premix *	**2.50**	**3.00**	**3.00**
L-Lysine HCl	**1.82**	**2.71**	**1.97**
Mycotoxin adsorbent	**1.00**	**1.00**	**1.00**
Anticoccidian	**0.50**	**0.50**	**0.50**
Antioxidant	**0.15**	**0.15**	**0.15**
Phytase	**0.08**	**0.08**	**0.08**
Total	1000.00	1000.00	1000.00
**Nutrients**	**Calculated analysis**		
Metabolizable energy (Kcal/Kg)	**3041**	**3100**	**3200**
Crude protein (%)	**22.5**	**21**	**20**
Non-phytic phosphorus (%)	**0.50**	**0.44**	**0.40**
Calcium (%)	**1.00**	**0.87**	**0.85**
Digestible lysine (%)	**1.27**	**1.15**	**1.00**
Digestible Methionine (%)	**0.61**	**0.58**	**0.52**
Digestible Met + Cis (%)	**0.98**	**0.87**	**0.80**
Digestible Threonine (%)	**-----**	**0.80**	**0.62**
Linoleic Acid (%)	**1.25**	**1.59**	**2.30**
Sodium (%)	**0.18**	**0.18**	**0.18**

* Vitamin A (12,000,000 IU), vitamin D3 (2,500,000 IU), vitamin E (15,000 IU), vitamin K (2.0 g), vitamin B1 (2.25 g), vitamin B2 (7.5 g), vitamin B6 (3.5 g), vitamin B12 (20 mg), folic acid (1.5 g), biotin (125 mg), pantothenic acid (12.5 g), niacin (45 g); Iron (50 g), zinc (50 g), manganese (110 g). copper (12 g), iodine (0.30 g), selenium (0.20 g), cobalt (0.20 g). Added amounts of vitamins and minerals per ton of feed.

**Table 2:** Productive performance of broilers at 42 days of age.

Treatment	Weight Gain (g)	Feed Intake (g)	Feed Conversion Ratio (kg × kg)	Mortality (%)
CON	2230.4 _a_	3789.3 _a_	1.70 _a_	2.50 _a_
ENR	2331.4 _b_	3853.8 _a_	1.65 _b_	6.00 _a_
PF	2326.4 _b_	3856.8 _a_	1.66 _b_	3.00 _a_
PF + EB	2308.0 _b_	3831.2 _a_	1.66 _b_	4.00 _a_
CPP	2312.6 _b_	3853.2 _a_	1.67 _b_	4.50 _a_
Probability	<0.0001	0.15	<0.0001	0.88
SEM	7.53	9.84	0.004	1.25

Different letters in the same column are statistically different (*p* < 0.05). SEM = Standard Error of the mean. Treatments: CON (Basal diet), ENR (Basal diet + Enradin^®^; 100 g/ton), PF (Basal diet + PF 300 g/ton), PF + EB (Basal diet + PF 250 g/ton; +EB 250 g/ton); CPP (Basal diet + CPP 300 g/ton).

**Table 3:** Average villus height and crypt depth in the duodenum, jejunum, and ileum of 42-day-old broilers fed diets supplemented with different eubiotics.

	Duodenum		Jejunum		Ileum	
Treatment	Villus Height	Crypt Depth	Villus Height	Crypt Depth	Villus Height	Crypt Depth
(µm)						
CON	2028.10 ^a^	293.07 ^a^	1091.19 ^a^	239.4 ^ab^	808.79 ^a^	200.10 ^ab^
ENR	2202.72 ^b^	287.02 ^a^	1159.52 ^ab^	225.68 ^a^	858.36 ^ab^	229.66 ^b^
PF	2254.12 ^b^	305.75 ^ab^	1209.98 ^bc^	244.71 ^ab^	869.97 ^ab^	189.58 ^a^
PF + EB	2227.41 ^b^	334.92 ^b^	1263.22 ^c^	244.88 ^ab^	909.13 ^b^	204.03 ^ab^
CPP	2221.30 ^b^	376.87 ^c^	1191.38 ^bc^	267.88 ^b^	864.88 ^ab^	209.95 ^ab^
Probability	0.001	<0.0001	<0.0001	0.024	0.036	0.006
SEM	18.701	5.370	9.900	4.104	10.079	3.527

Different letters in the same column are statistically different (*p* < 0.05). SEM = standard error of the mean. Treatments: CON (Basal diet), ENR (Basal diet + Enradin^®^; 100 g/ton), PF (Basal diet + PF 300 g/ton), PF + EB (Basal diet + PF 250g/ton; +EB 250g/ton); CPP (Basal diet + CPP 300 g/ton).

**Table 4:** Average ratio of villus height/crypt depth in the duodenum, jejunum, and ileum of 42-day-old broilers fed diets supplemented with different eubiotics.

Treatment	Duodenum	Jejunum	Ileum
CON	7.10 ^ab^	4.68 ^a^	4.09 ^ab^
ENR	7.79 ^b^	5.19 ^ab^	3.82 ^a^
PF	7.97 ^b^	5.14 ^ab^	4.85 ^c^
PF + EB	6.89 ^ab^	5.40 ^b^	4.56 ^bc^
CPP	6.03 ^a^	4.69 ^a^	4.25 ^abc^
Probability	<0.0001	0.019	<0.0001
SEM	0.151	0.085	0.080

Different letters in the same column are statistically different (*p* < 0.05). SEM = standard error of the mean. Treatments: CON (Basal diet), ENR (Basal diet + Enradin^®^; 100 g/ton), PF (Basal diet + PF 300 g/ton), PF + EB (Basal diet + PF 250 g/ton; +EB 250 g/ton); CPP (Basal diet + CPP 300 g/ton).

**Table 5:** Average intestinal mucosal thickness in the duodenum, jejunum, and ileum of 42-day-old broilers fed diets supplemented with different eubiotics.

Treatment	Duodenum	Jejunum	Ileum
CON	2321.17 ^a^	1330.64 ^a^	1015.55 ^a^
ENR	2489.74 ^b^	1385.21 ^ab^	1101.37 ^ab^
PF	2559.88 ^b^	1454.69 ^bc^	1059.55 ^ab^
PF + EB	2562.33 ^b^	1478.10 ^c^	1106.49 ^b^
CPP	2534.84 ^b^	1452.59 ^bc^	1074.82 ^ab^
Probability	<0.0001	<0.0001	0.037
SEM	19.223	10.026	10.370

Different letters in the same column are statistically different (*p* < 0.05). SEM = Standard Error of the Mean. Treatments: CON (Basal diet), ENR (Basal diet + Enradin^®^; 100 g/ton), PF (Basal diet + PF 300 g/ton), PF + EB (Basal diet + PF 250 g/ton; +EB 250 g/ton); CPP (Basal diet + CPP 300 g/ton).

**Table 6:** Average carcass yield of 42-day-old broilers fed diets supplemented with different eubiotics.

Treatment	Carcass Yield (%)	Carcass Weight (g)	Carcass Yield (%)	Carcass Weight (g)
Hot			Chilled	
CON	71.3 ^a^	1746.1 ^a^	72.4 ^a^	1773.2 ^a^
ENR	72.0 ^a^	1885.8 ^b^	73.1 ^a^	1915.2 ^b^
PF	72.3 ^a^	1888.5 ^b^	73.4 ^a^	1918.0 ^b^
PF + EB	72.2 ^a^	1886.7 ^b^	73.1 ^a^	1910.5 ^b^
CPP	71.7 ^a^	1860.9 ^b^	72.6 ^a^	1883.8 ^b^
Probability	0.06	0.002	0.5	0.002
SEM	0.12	14.27	0.12	14.31

Different letters in the same column are statistically different (*p* < 0.05). SEM = Standard Error of the Mean. Treatments: CON (Basal diet), ENR (Basal diet + Enradin^®^; 100 g/ton), PF (Basal diet + PF 300 g/ton), PF + EB (Basal diet + PF 250 g/ton; +EB 250 g/ton); CPP (Basal diet + CPP 300 g/ton).

**Table 7:** Average skin pigmentation of 42-day-old broilers fed diets supplemented with different eubiotics.

	Redness of the Skin (a*)		Yellowness of the Skin (b*)	
Treatment	In Vivo	Carcass	In Vivo	Carcass
CON	1.87 ^a^	2.68 ^a^	19.57 ^a^	40.23 ^a^
ENR	1.80 ^a^	3.70 ^ab^	20.27 ^a^	40.54 ^a^
PF	2.49 ^a^	4.63 ^b^	18.76 ^a^	39.57 ^a^
PF + EB	1.87 ^a^	3.05 ^ab^	19.53 ^a^	41.61 ^a^
CPP	1.89 ^a^	2.18 ^a^	18.85 ^a^	40.70 ^a^
Probability	0.39	0.006	0.25	0.27
SEM	0.12	0.24	0.24	0.29

Different letters in the same column are statistically different (*p* < 0.05). SEM = Standard Error of the Mean. Treatments: CON (Basal diet), ENR (Basal diet + Enradin^®^; 100 g/ton), PF (Basal diet + PF 300 g/ton), PF + EB (Basal diet + PF 250 g/ton; +EB 250 g/ton); CPP (Basal diet + CPP 300 g/ton).

## Data Availability

The datasets generated during and/or analyzed during the study are available from the corresponding author upon reasonable request.
